# Epithelial morphogenesis in the *Drosophila* egg chamber requires Parvin and ILK

**DOI:** 10.3389/fcell.2022.951082

**Published:** 2022-12-01

**Authors:** Athina Keramidioti, Evgenia Golegou, Eleni Psarra, Nikolaos Paschalidis, Konstantina Kalodimou, Shinya Yamamoto, Christos Delidakis, Katerina M. Vakaloglou, Christos G. Zervas

**Affiliations:** ^1^ Center of Basic Research, Biomedical Research Foundation, Academy of Athens, Athens, Greece; ^2^ Department of Biochemistry and Biotechnology, University of Thessaly, Larissa, Greece; ^3^ Department of Molecular and Human Genetics, Department of Neuroscience (BCM), The Development Disease Models and Therapeutics Graduate Program, Baylor College of Medicine (BCM), Texas Children’s Hospital (TCH), Program in Developmental Biology (BCM), Jan and Dan Duncan Neurological Research Institute, Houston, TX, United States; ^4^ Department of Biology, University of Crete, Iraklio, Greece; ^5^ Foundation for Research and Technology-Hellas (FORTH), Institute of Molecular Biology and Biotechnology (IMBB), Iraklio, Greece

**Keywords:** oogenesis, integrin, cell adhesion, cytoskeleton, germline cyst encapsulation

## Abstract

Integrins are the major family of transmembrane proteins that mediate cell-matrix adhesion and have a critical role in epithelial morphogenesis. Integrin function largely depends on the indirect connection of the integrin cytoplasmic tail to the actin cytoskeleton through an intracellular protein network, the integrin adhesome. What is currently unknown is the role of individual integrin adhesome components in epithelia dynamic reorganization. *Drosophila* egg chamber consists of the oocyte encircled by a monolayer of somatic follicle epithelial cells that undergo specific cell shape changes. Egg chamber morphogenesis depends on a developmental array of cell-cell and cell-matrix signalling events. Recent elegant work on the role of integrins in the *Drosophila* egg chamber has indicated their essential role in the early stages of oogenesis when the pre-follicle cells assemble into the follicle epithelium. Here, we have focused on the functional requirement of two key integrin adhesome components, Parvin and Integrin-Linked Kinase (ILK). Both proteins are expressed in the developing ovary from pupae to the adult stage and display enriched expression in terminal filament and stalk cells, while their genetic removal from early germaria results in severe disruption of the subsequent oogenesis, leading to female sterility. Combining genetic mosaic analysis of available null alleles for both *Parvin* and *Ilk* with conditional rescue utilizing the UAS/Gal4 system, we found that Parvin and ILK are required in pre-follicle cells for germline cyst encapsulation and stalk cell morphogenesis. Collectively, we have uncovered novel developmental functions for both Parvin and ILK, which closely synergize with integrins in epithelia.

## Introduction

**FIGURE 1:**
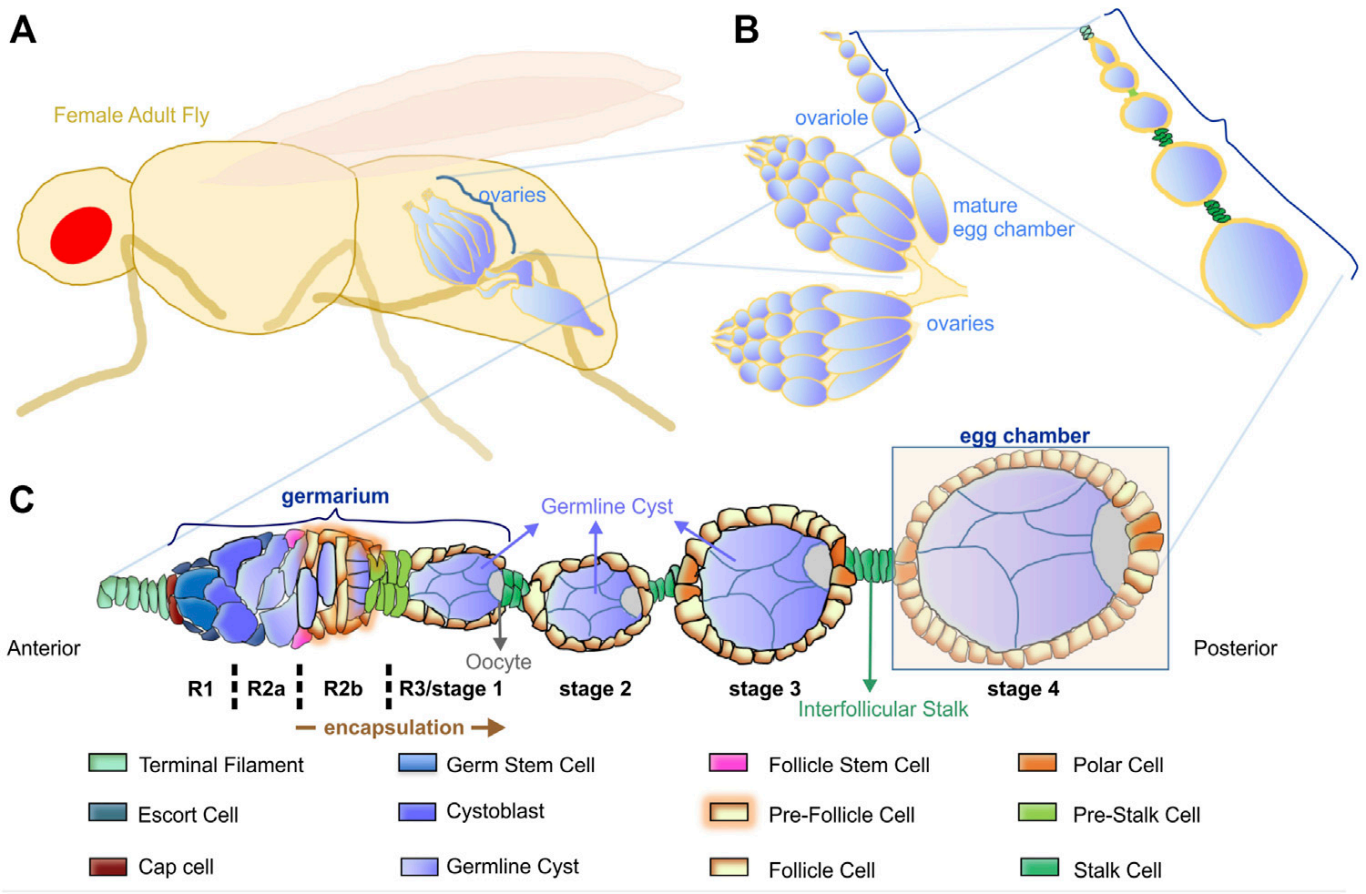
Overview of *Drosophila* oogenesis. **(A)** The female adult fly has a pair of ovaries. **(B)** Each ovary comprises 16–20 ovarioles that contain linearly arranged egg chambers of increasing age. **(C)** Schematic representation of the cellular organization of the germarium and the consecutive egg chambers. The germarium is located at the anterior tip, where germ cells continuously divide and initiate oogenesis. The germarium is subdivided into four distinct morphological regions (R1, R2a, R2b, R3/stage1). At the posterior tip of the germarium, the pre-follicle cells complete the encapsulation of each cyst and form the egg chamber. An interfollicular stalk separates the egg chambers of different developmental stages.

Collective cell movement and cell intercalation are two essential morphogenetic processes that direct tissue formation (Walck-Shannon and Hardin, 2014; Scarpa and Mayor, 2016). *Drosophila* oogenesis possesses a full repertoire of morphogenetic procedures such as collective cell movement during egg chamber encapsulation (Margolis and Spradling, 1995; Sang, 1970) and cell intercalation during interstitial stalk formation (Godt and Laski, 1995). The beauty of the *Drosophila* egg chamber as a model system in developmental biology lies in its rather simple but well-defined anatomical and cellular organization that can be combined with genetic analysis (Bilder and Haigo, 2012). The egg chamber is composed of a follicle epithelium that encircles the developing oocyte (Figure 1). The onset of follicle morphogenesis is initiated in the dorsal and ventral side at the posterior part of the germarium, where a small number of follicle stem cells (FSCs) that are adult stem cells give rise to the precursors of follicle epithelial cells (Fadiga and Nystul, 2019), (Figure 1C). After four to six rounds of cell divisions, these precursors produce approximately 1,000 cells that assemble into the follicle epithelium monolayer, which enlarges and elongates to finally produce the mature egg (Duhart et al., 2017). Meanwhile, at the tip of the germarium, germline stem cells (GSCs) divide asymmetrically. One cell remains a stem cell, while the other differentiates to a cystoblast. After four mitotic divisions, the cystoblast transforms into a 16-cells syncytium. During mitotic divisions, the cyst moves towards region two of the germarium. The 16-cells cyst is encapsulated by the pre-follicle cells in region 2b (Figure 1C). During cyst encapsulation, the pre-follicle cells extend thin centripetal processes around the newly formed germline cyst to separate it from its neighbours. Next, several somatic follicle epithelial cells migrate around the new cyst, separating it from its younger and older neighbours by an epithelial monolayer. The 16-cells cyst now is called germline cyst and covers the diameter of the germarium (Figure 1C) and (Finegan and Bergstralh, 2020).

Stalk cells, through the morphogenetic process of intercalation, form the interfollicular stem that connects the successive egg chambers and defines the anterior-posterior long axis of the developing cyst (Roth and Lynch, 2009). The stem originates as a double row of elongated cells in regions 2b to 3, which intercalate resulting in a single row of cells at later stages (Godt and Laski, 1995). Stalk cells are also responsible for the proper localization of the oocyte in the younger cyst. The stalk cells in direct contact with the young cyst up-regulate DE-cadherin in the posterior follicle cells of the cyst. Furthermore, an increase of DE-cadherin levels in the oocyte of the young cyst leads to its adhesion to the posterior follicle cells obtaining from now on a posterior localization and establishing the anteroposterior axis of the cyst (Godt and Tepass, 1998; Gonzales-Reyes and St. Johnston, 1998; Bécam et al., 2005).

**FIGURE 2:**
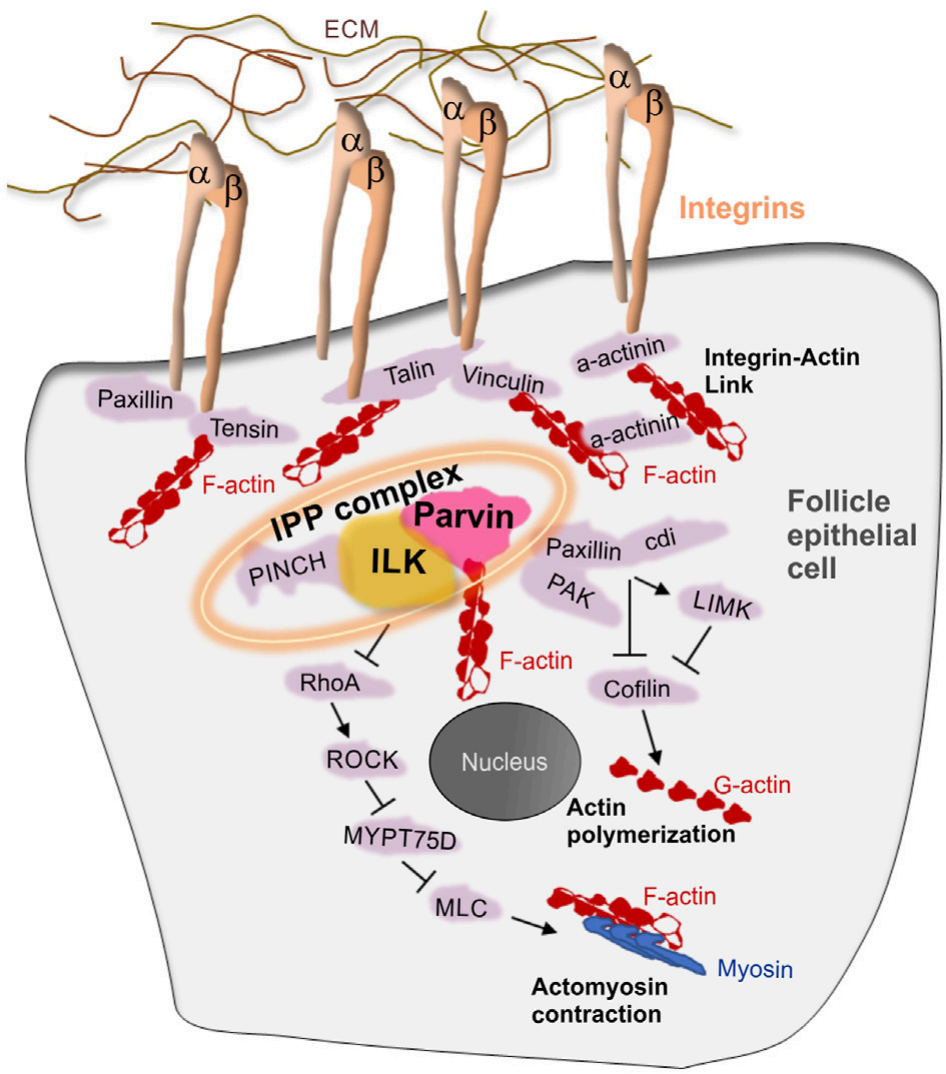
**The integrin adhesome in the *Drosophila* follicle epithelium.** Schematic representation depicting a model of integrin-associated proteins in the follicle epithelium. Several integrin adhesome proteins have been identified to express and function in the follicle cells. The tripartite IPP complex is a conserved protein complex containing Integrin-Linked Kinase (ILK), Parvin and PINCH and plays central role in the assembly and function of the integrin adhesome.

Previous work has demonstrated the essential role of integrins in follicle epithelium morphogenesis (Bolivar et al., 2006; Fernández-Miñán et al., 2007; Fernández-Miñán et al., 2008; Gómez-Lamarca et al., 2014; Lovegrove et al., 2019; Van De Bor et al., 2021). Loss of β_PS_ integrin subunit -encoded by the *myospheroid* locus-in the germarium fails cyst formation (Bolivar et al., 2006), disrupts the monolayered epithelium organization at egg chamber termini and the assembly of the interfollicular stalks between adjacent egg chambers, leading to a fused egg chamber phenotype (Fernández-Miñán et al., 2007; Gómez-Lamarca et al., 2014; Lovegrove et al., 2019; Van De Bor et al., 2021). Despite the importance of integrins in follicle epithelial morphogenesis, very little information is known about the integrin adhesome proteins that function downstream of integrins in early stages of egg chamber development (Figure 2). However, several of the integrin adhesome proteins have been identified to cooperate with integrins at later developmental stages of oogenesis to promote egg chamber elongation (Baum and Perrimon, 2001; Chen et al., 2001; Chen et al., 2005; Wahlström et al., 2006; Delon and Brown, 2009; He et al., 2010; Sun et al., 2011; Kelpsch et al., 2016; Maartens et al., 2016; Cha et al., 2017; Qin et al., 2017). Parvin and Integrin-linked kinase (ILK) are two central components of the integrin adhesome in both mammals and flies (Winograd-Katz et al., 2014; Green and Brown, 2019). We have previously shown that both *Parvin* and *Ilk* are essential to mediate the stable adhesion of integrins to the extracellular matrix (ECM) at the muscle attachment sites in the fly embryo (Vakaloglou et al., 2016). Here we examine the role of both Parvin and ILK in early oogenesis, as a means to identify the molecular machinery that modulates integrin-mediated adhesion in the developing epithelium. We demonstrate that Parvin and ILK are necessary for germline cyst encapsulation, egg chamber separation, oocyte positioning and assembly of the interfollicular stalk. These findings highlight the pivotal novel role of Parvin and ILK in epithelial morphogenesis during egg chamber development and provide a new model system to dissect the molecular mechanism of their *in vivo* functions.

## Results

### Parvin-GFP and ILK-GFP coexpress and colocalize in the developing ovary

**FIGURE 3:**
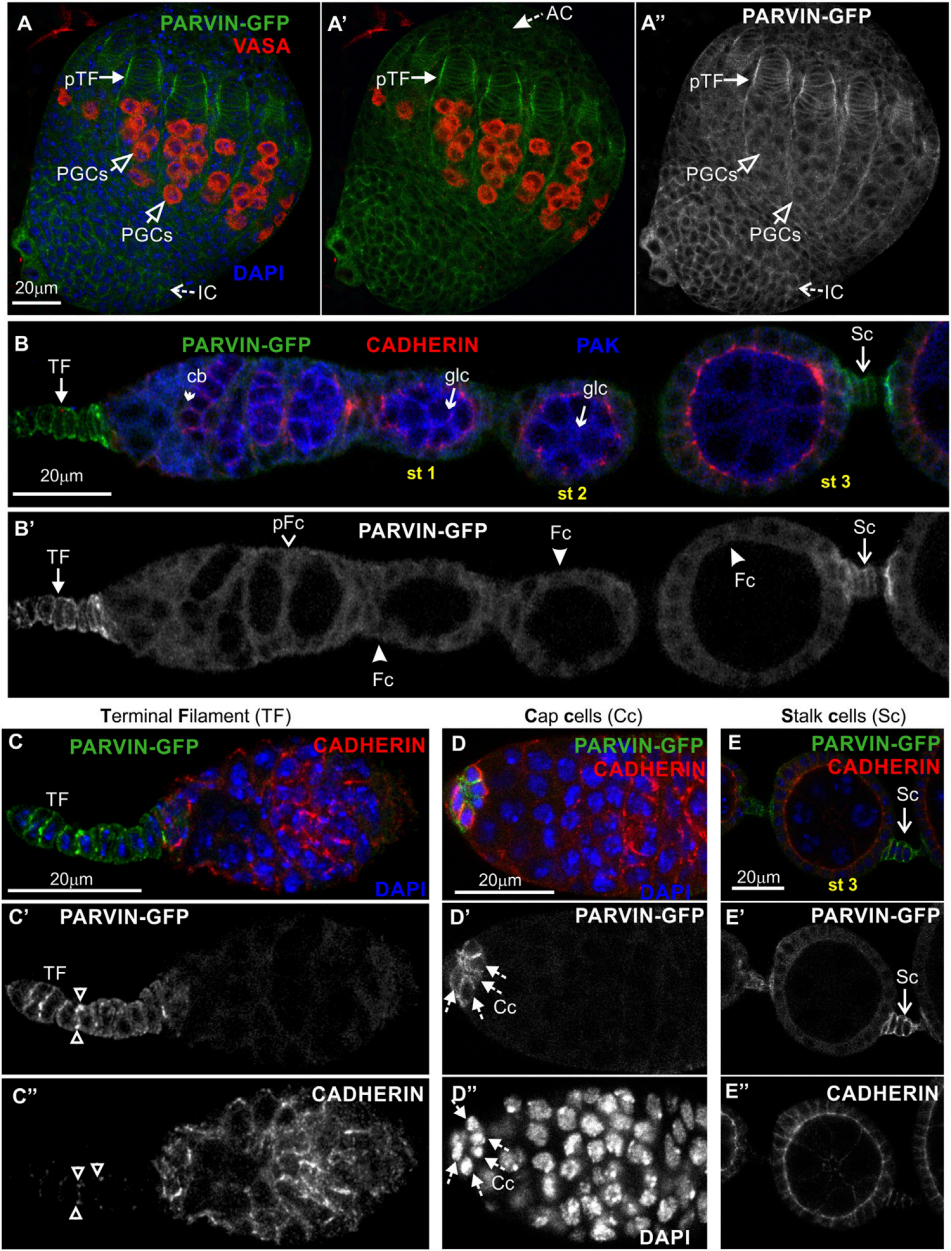
Parvin-GFP expression in the developing ovary and the adult egg chamber. Confocal micrographs of a pupal ovary and adult egg chambers, all expressing GFP-tagged Parvin to reveal the endogenous spatiotemporal protein expression and subcellular distribution. **(A-A")** Early pupal (2–4h APF) ovary expressing Parvin-GFP. The protein is enriched in the lateral sites of terminal filament cell precursors and in the periphery of interstitial cells, apical cells and primordial germ cells. PGCs were marked with antibodies against Vasa. **(B–E")** Parvin-GFP expression in the early previtellogenic egg chambers of an adult ovariole shows (B-B′) low level of protein expression in pre-follicle and follicle cells, but significant enrichment in the periphery of **(C-C")** terminal filament cells, **(D-D")** cap cells in the germarium region and **(E-E")** in the interfollicular stalk cells. Co-staining against Cadherin and PAK proteins shows for Parvin-GFP a clearly distinct pattern of expression and localization from the epithelial and germ cell lineages. AC, apical cells; pTF, precursor of terminal filament; TF, terminal filament; PGCs, primordial germ cells; IC, interstitial cells; Cc, cap cells; cb, cystoblast; glc, germline cyst; pFC, pre-follicle cell; Fc, follicle cells; Sc, stalk cells. Scale bars: 20 μm in all panels.

**FIGURE 4:**
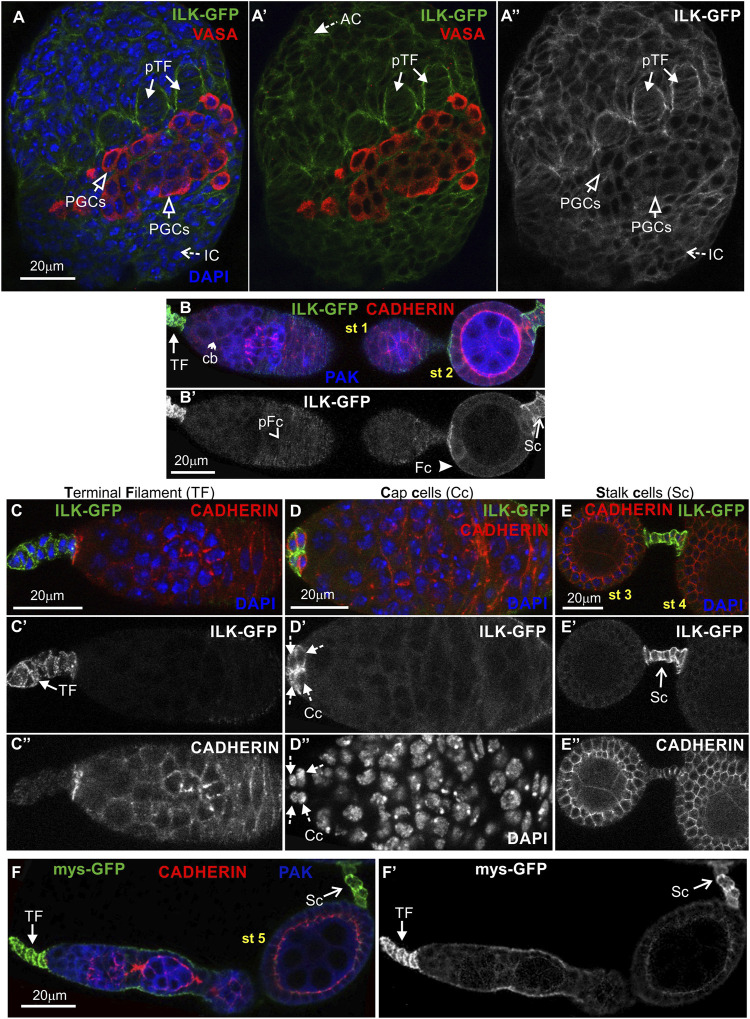
ILK-GFP expression in the developing ovary and the adult egg chamber. Confocal micrographs of ovarioles and egg chambers expressing GFP-tagged ILK and β_PS_ integrin (*mys*) reveal their endogenous protein expression and subcellular distribution. **(A-A")** Early pupal (2–4h APF) ovary expressing ILK-GFP. The protein is enriched in the lateral sites of terminal filament cell precursors and in the periphery of interstitial cells, apical cells and primordial germ cells. PGCs were marked with antibodies against Vasa. **(B-B')** ILK-GFP expression in the early previtellogenic egg chambers of an adult ovariole shows **(B-B′)** low level of protein expression in pre-follicle and follicle cells, but significant enrichment in the periphery of **(C-C")** terminal filament cells, **(D-D")** cap cells in the germarium region and **(E-E")** in the interfollicular stalk cells. Co-staining against Cadherin and PAK proteins shows for ILK-GFP a clearly distinct pattern of expression and localization from the epithelial and germ cell lineages. **(F-F′)** *mys*-*GFP* is highly accumulated in terminal filament and stalk cells, similar to Parvin-GFP and ILK-GFP. Each image is representative of at least five different imaged ovarioles of the same genotype and markers used. AC, apical cells; pTF, precursor of terminal filament; TF, terminal filament; PGCs, primordial germ cells; IC, interstitial cells; Cc, cap cells; cb, cystoblast; glc, germline cyst; pFC, pre-follicle cells; Fc, follicle cells; Sc, stalk cells. Scale bars: 20 μm in all panels.

To analyze Parvin and ILK functions in the developing *Drosophila* ovary, we first characterized the expression pattern of both proteins by examining ovaries from transgenic fly strains expressing genomic translational fusion rescue constructs for each gene tagged with GFP (Zervas et al., 2001; Vakaloglou et al., 2012). Initially, we examined ovaries at the early pupal stage (2–4 h APF: After Pupae Formation) where the apical cells differentiate on epithelial sheath cells (Reilein et al., 2021), start migrating to the base of the developing ovary and secrete ECM molecules on their basal side delimiting in this way the newly formed ovarioles. Both Parvin-GFP and ILK-GFP were evident in the cytoplasm of both somatic cells and primordial germ cells (PGCs) labelled with Vasa (Figure 3A-A″; Figure 4A-A″). In the precursors of terminal filament cells, Parvin-GFP and ILK-GFP were enriched at the lateral sites of the cells, which are in contact with the respective cells of adjacent columns (Figure 3A′′; Figure 4A″).

In the adult ovary, Parvin-GFP and ILK-GFP displayed also a similar expression pattern in the early stage egg chambers (Figure 3B-B′; Figure 4B-B′). Both proteins were expressed at low levels in pre-follicle and follicle cells (Parvin-GFP: follicle cells, 16.89 ± 3.85 mean grey values; Parvin-GFP: pre-follicle cells, 23.59 ± 6.69 mean grey values; ILK-GFP: follicle cells, 9.33 ± 2.86 mean grey values; ILK-GFP: pre-follicle cells, 11.39 ± 5.37 mean grey), where they appeared diffuse in the cytoplasm, clearly distinct from the cadherin-labelled lateral and apical side (Figure 3B; Figure 4B). Interestingly, Parvin-GFP and ILK-GFP were enriched in terminal filament cells (Figure 3C-C′; Figure 4C-C′) (Parvin-GFP: 50.16 ± 16.16 mean grey values; ILK-GFP:85.58 ± 11.53), cap cells (Figure 3D-D′; Figure 4D-D′) (Parvin-GFP: 38.24 ± 15.27 mean grey values; ILK -GFP: 62.45 ± 14.13 mean grey values) and stalk cells (Figure 3E-E′; Figure 4E-E′) (Parvin-GFP: 41.26 ± 8.04 mean grey values; ILK -GFP: 41.26 ± 22.83 mean grey values) (Figures 3C–E; Figures 4C–E). We finally verified the expression pattern of the β_PS_ integrin subunit, by examining ovaries from a *mys-GFP* strain (Klapholz et al., 2015). *Mys-GFP* was strongly expressed in the terminal filament cells of the germarium and differentiated stalk cells (Figure 4F-F′), similar to Parvin-GFP and ILK-GFP.

Overall, Parvin-GFP and ILK-GFP display an identical expression pattern in the *Drosophila* ovary, which fits with their property to act as a complex (Wickstrom et al., 2010). Also, both proteins exhibit major similarities with the expression pattern of *mys-GFP* in the adult ovariole.

### Parvin and ILK are required for female fecundity

**FIGURE 5:**
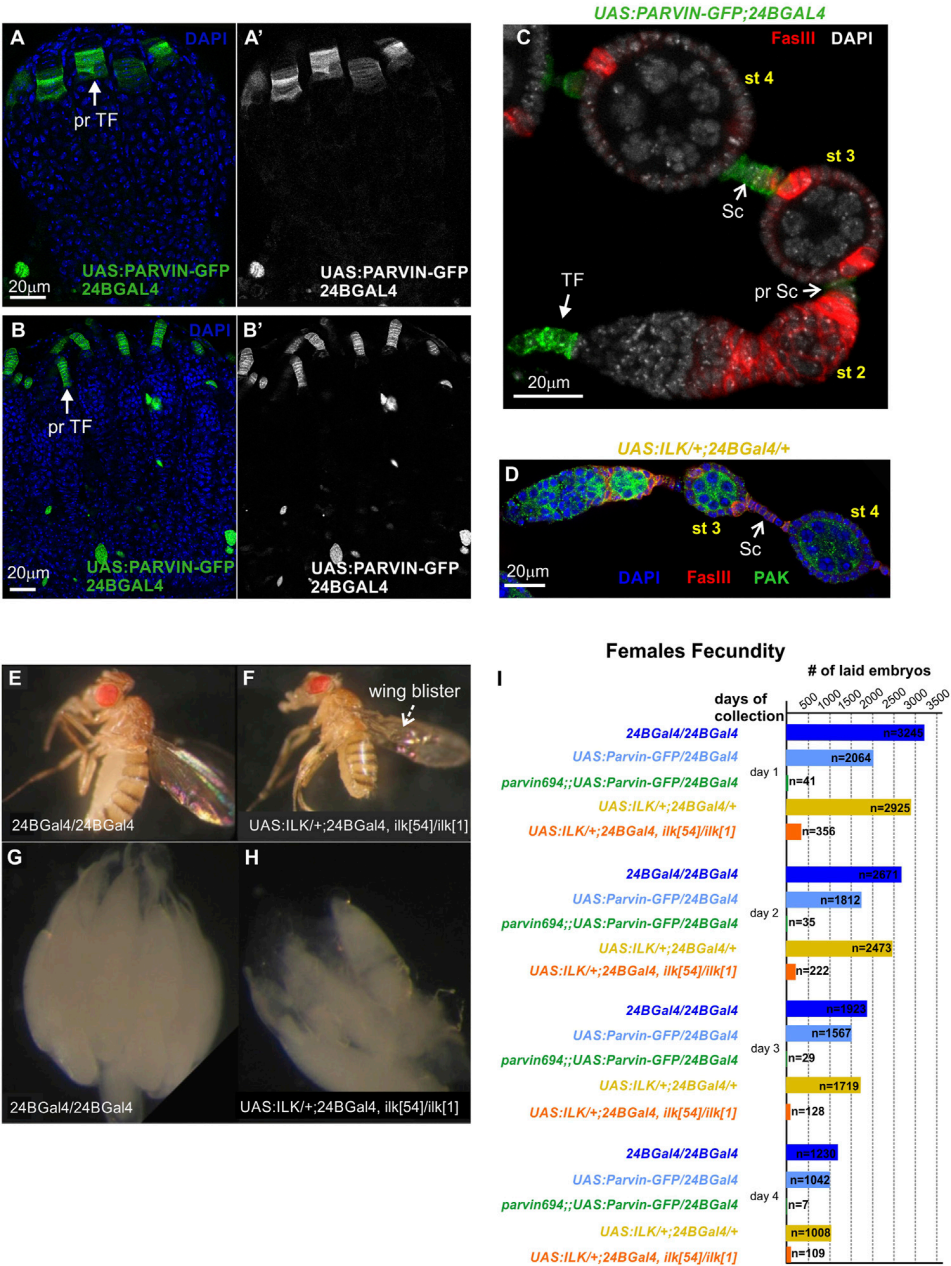
Parvin and ILK are required for female fecundity. **(A-B′)** Ovaries from **(A)** early pupal stage and **(B)** pharate adult. *24BGal4* is expressed in the precursors of terminal filament cells in the **(A-A′)** early pupal stage and **(B-B′)** pharate adult. **(C,D)** *24BGal4* expression pattern in adult ovary. *24BGal4*-mediated expression of **(C)** Parvin-GFP and **(D)** ILK, in terminal filament cells, in pre-stalk and in fully differentiated stalk cells does not affect the structure and morphology of the developing ovariole. **(E)** *24BGal4* adult fly and **(F)** *24BGal4*-rescued *Ilk* mutant adult fly with wing blisters due to lack of *24BGal4* expression in the wing epithelium. **(G–H)** Ovaries from **(G)** *24BGal4* adult fly, **(H)** *24BGal4*-rescued *Ilk* mutant adult fly. The later appears smaller in size and contains a decreased number of mature egg chambers. Each image is representative of at least ten different imaged adult flies, five different imaged ovaries or five different imaged ovarioles of the same genotype and markers used. **(I)** Graphic illustration of the obtained results in the female fecundity assay. The *24BGal4*-mediated rescued adult flies for either *Parvin* or *Ilk* null alleles laid a significantly reduced number of embryos compared to controls (flies homozygous for *24BGal4,* or expressing a single copy of *UAS:Parvin-GFP* and *24BGal4*, or expressing a single copy of UAS:ILK and *24BGal4* respectively). pr TF, precursors of terminal filament; TF, terminal filament; pr Sc, pre-stalk cells; Sc, stalk cells. Scale bars: 20 μm in all panels.

Both *Parvin* and *Ilk* are essential genes and null *Drosophila* homozygous mutants die at the end of embryogenesis (Zervas et al., 2001; Vakaloglou et al., 2012). Thus, to circumvent the embryonic lethality associated with *Parvin* and *Ilk* mutations and identify novel functions, we took advantage of the UAS/Gal4 system. We previously have shown that *Parvin* or *Ilk* null mutations can be rescued to adult viability by expressing one copy of the corresponding UAS transgene by *24BGal4* (Zervas et al., 2001; Vakaloglou et al., 2012). *24BGal4* is expressed in the mesodermally derived tissues from early embryogenesis (Brand and Perrimon, 1993). Because somatic gonadal precursors have a mesodermal lineage, we initially verified that *24BGal4* is expressed in the precursors of terminal filament cells in the early pupae stage (Figure 5A-A′) and in the pharate adult (Figure 5B-B′). In the adult ovariole, *24BGal4* is expressed in terminal filament cells and in fully differentiated stalk cells at stages 2–3 (Figure 5C). We further identified that the induced levels of either UAS:Parvin-GFP or UAS:ILK do not affect the structure and morphology of the differentiated follicle epithelium highlighted by FasIII (Figures 5C,D) and result in fertile flies (Figures 5C,D,I). Thus, we could use the conditionally rescued female adult flies to analyze the functional requirement of Parvin and ILK in oogenesis. *24BGal4*-rescued adult flies for either *Parvin* (*Parvin* ^*694*^/*Parvin* ^*694*^ *;;UAS:Parvin-GFP/24B Gal4*) or *Ilk* (*UAS:ILK/+;;ilk* ^*54*^ *, FRT2A/ilk* ^*1*^ *, 24BGal4*) had blisters in both wings due to lack of *24BGal4* expression in the wing epithelial cells (Figures 5E,F) (Zervas et al., 2001). We then examined the morphology of ovaries derived from the *24BGal4* rescued adult flies. These ovaries were smaller in size and contained a reduced number of mature egg chambers in comparison to the *24BGal4* homozygous flies (Figures 5G,H). We then measured the fecundity in the *Parvin* and *Ilk* conditionally rescued female adults. We used three genotypes of flies as controls in our measurements. The first one expresses two copies of *24BGal4*, the second expresses one copy of *UAS:Parvin-GFP* and one copy of *24BGal4* and the third expresses one copy of *UAS:ILK* and one copy of *24BGal4* (Figure 5I). The same number of adult females was used for each comparison and we counted the number of embryos laid in four consecutive days. We first found that the moderately expressing *UAS:Parvin-GFP* lines that we used, caused a significant reduction in embryos laid at 1 and 2 days (Figure 5I). Because we have previously shown that highly expressing *UAS:Parvin-GFP* lines driven by *24BGal4* result in lethality while moderate expression levels in Parvin-GFP lines do not (Chountala et al., 2012), we cannot exclude the possibility that even moderate levels of Parvin-GFP expression may reduce the fly fitness resulting in the reduced ability of female flies to lay embryos in the first 2 days. However, *Parvin* and *Ilk 24BGal4* rescued female adult mutants laid extremely low numbers of embryos, which were decaying and never hatched (Figure 5I). Thus, we concluded that both Parvin and ILK are required in the somatic epithelium and their expression only in terminal filament and stalk cells, where *24BGal4* is expressed, is not sufficient to promote proper egg chamber development and restore female fertility.

### Parvin and ILK are required in the pre-follicle epithelial cells to facilitate germline cyst encapsulation and stalk morphogenesis

**FIGURE 6:**
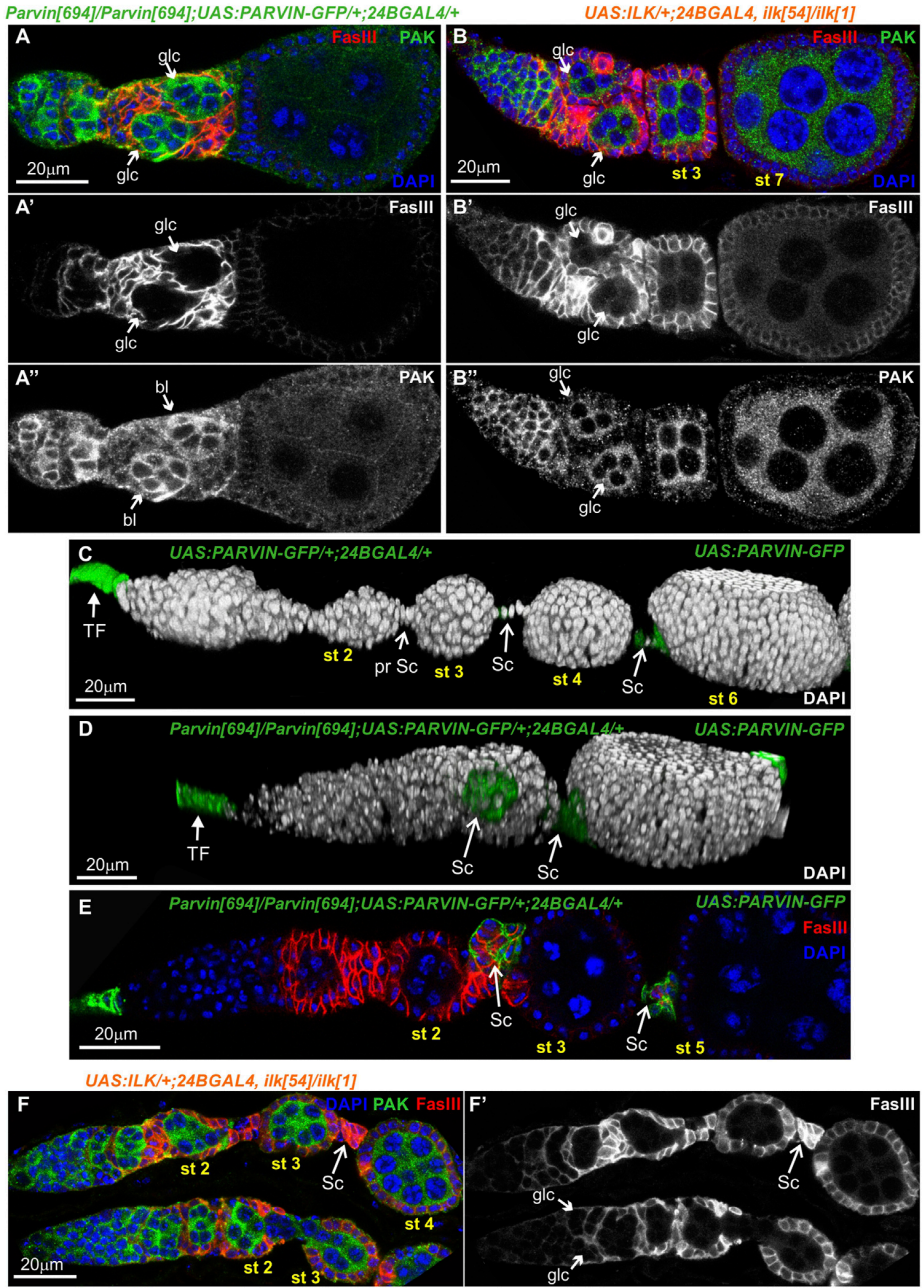
Parvin and ILK are required in the pre-follicle epithelial cells to facilitate germline cyst encapsulation and interfollicular stalk morphogenesis. *24BGal4*-rescued adult mutant flies for either *Parvin* or *Ilk* null alleles exhibit germline cyst and stalk organization defects in the germaria. **(A-B")** Confocal micrographs of ovarioles and egg chambers derived from *24BGal4*-rescued adult flies for **(A-A")** *Parvin* and **(B-B")** *Ilk* contain two cysts in the region 2b of the germarium. **(C,D)** 3D rendering of representative ovarioles derived from **(C)** adult flies that express one copy of *UAS:Parvin-GFP* under the *24BGal4* driver and exhibit a wild type morphology for the ovariole and **(D)** *24BGal4*-rescued adult mutant flies for *Parvin* null allele, carrying clusters of stalk cells in the ovariole. Stalk cells were identified by the expression of Parvin-GFP driven by the *24BGal4*. **(E,F)** Cross-sections of ovarioles of *24BGal4*-mediated rescued adult flies for either *Parvin* **(E)** or *Ilk* **(F)** showing the stalk cell clusters in between stage 2 (st2) and 3 (st3) egg chambers. glc, germline cyst; TF, terminal filament; Sc, Stalk cell. Scale bars: 20 μm in all panels.

To further characterize the morphological defects in the developing egg chambers associated with the lack of either Parvin or ILK from the somatic epithelial cells, we dissected and stained ovaries with a variety of cellular markers. We initially identified in a large fraction of *24BGal4* rescued *Parvin* ^*694*^ (n = 61/89) (Figure 6A-A″), or *Ilk* ^*54*^ (*n* = 25/41) (Figure 6B-B″) dissected ovarioles, the germaria in region 2b contained two germline cysts rather than a single one. However, each of these cysts of comparable shape and size was fully encircled by pre-follicle cells, so the gamete cells remained rather separated (Figures 6A,B). Thus, we concluded that both Parvin and ILK are required in the encapsulation process at the germarium stage. We then examined the stalk cell organization (Figures 6C–F). Stalks, usually comprise of four to eight cells assembled as a single cell layer that separate neighbouring egg chambers (Figure 5C; Figure 6C; Supplementary Figure S1). Egg chambers in ovarioles derived from the *24BGal4* rescued *Parvin* ^*694*^ (*n* = 89/89), or *Ilk* ^*54*^ (*n* = 41/41) mutant female flies had always defective interfollicular stalks. Approximately half of the egg chambers (*n* = 46/89 in *Parvin* ^*694*^ rescued; *n* = 26/41 in *Ilk* ^*54*^ rescued) carry an ectopically positioned cluster of stalk cells, which have not been properly intercalating but maintain association with the consecutive egg chambers (Figures 6D,E; Supplementary Figure S1). In a fraction of these defective egg chambers we were able to accurately quantify the number of cells forming the cluster and found that contained a higher number of stalk cells compared to the control genotypes (Supplementary Figure S2). In the UAS:Parvin-GFP/*24BGal4* rescued *Parvin* ^*694*^ the identification of the stalk cells was based on the expression of UAS:Parvin-GFP. In contrast, in the UAS:ILK/*24BGal4* rescued *Ilk* ^*54*^ the identification of the stalk cells was based on FasIII expression and relative position in the egg chamber (Figure 6F-F′).

### Genetic mosaics for parvin and ilk confirm their essential role in germline cyst encapsulation

**FIGURE 7:**
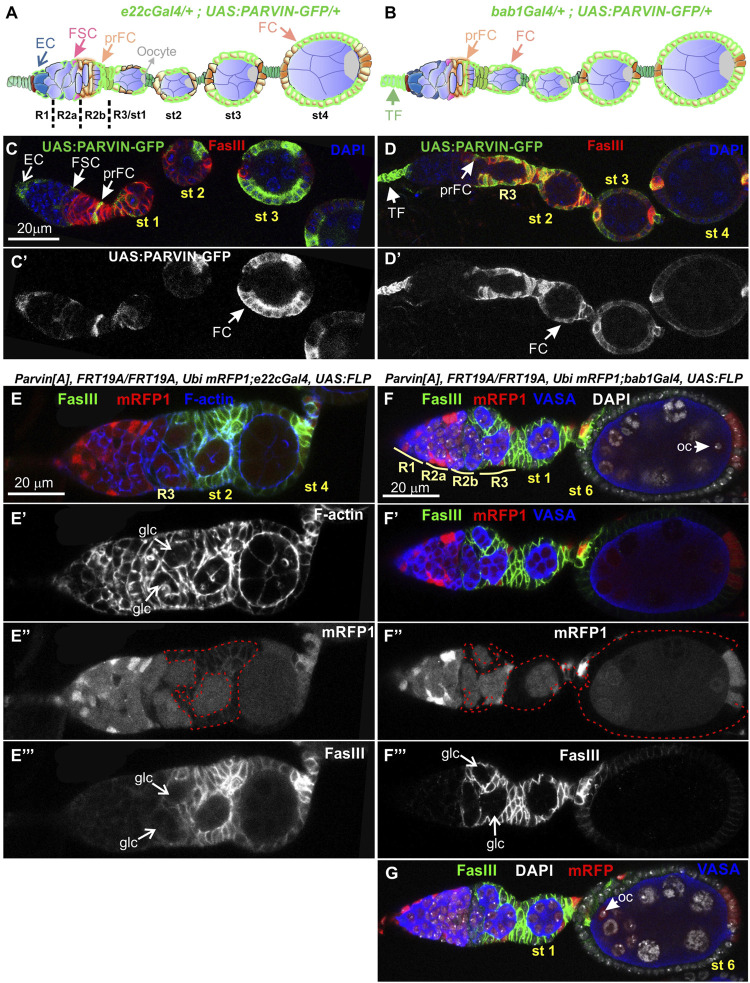
Genetic mosaics for *Parvin* display germline cyst encapsulation defects resulting in compound egg chambers. **(A,B)** Graphic illustration and expression pattern characterization in the egg chambers of **(A)** *e22cGal4* and **(B)** *bab1Gal4*, derived from adult flies expressing UAS:Parvin-GFP. **(C,D)** Confocal micrographs illustrate that **(C-C′)** *e22cGal4* is expressed in the escort cells in the anterior part of the germarium, in the follicle stem cells at the interface between regions 2a and 2b and in almost all follicle epithelial cells after stage 2. **(D-D′)** *bab1Gal4* is expressed in terminal filament cells in the anterior part of the germarium, in the pre-follicle cells of the germarium, in polar cells and in a large fraction of follicle epithelial cells after stage 2. **(E-E‴)**, **(F-F‴)**, **(G)** Confocal micrographs of egg chambers derived from *Parvin* ^*A*^ mosaic flies generated by **(E-E‴)** *e22cGal4* or **(F-F‴)**, **(G)** *bab1Gal4*. **(E"-F")** *Parvin* ^*A*^ mutant cells were marked by the absence of *mRFP1* and are highlighted with a red dashed line. Two cysts in region 2b are enclosed in *Parvin* ^*A*^ mosaic egg chambers generated with either of the *Gal4* drivers **(F)** and **(G)** are different optical sections of the same egg chamber to reveal the compound egg chamber presence at stage 6, which contains two oocyte nuclei. The oocyte nuclei are located in the posterior **(F)** and the anterior **(G)** end of the egg chamber containing a large *Parvin* ^*A*^ mutant clone. **(E′,F′)** F-actin and **(E‴,F‴)** FasIII label the periphery of the follicle epithelial cells, while Vasa labels the germ cells. EC, Escort Cell; FSC, Follicle Stem Cell; TF, Terminal Filament; pr FC, pre-Follicle Cell; FC, Follicle Cell; Oc, Oocyte; glc, germline cyst. Scale bars: 20 μm in all panels.

**FIGURE 8:**
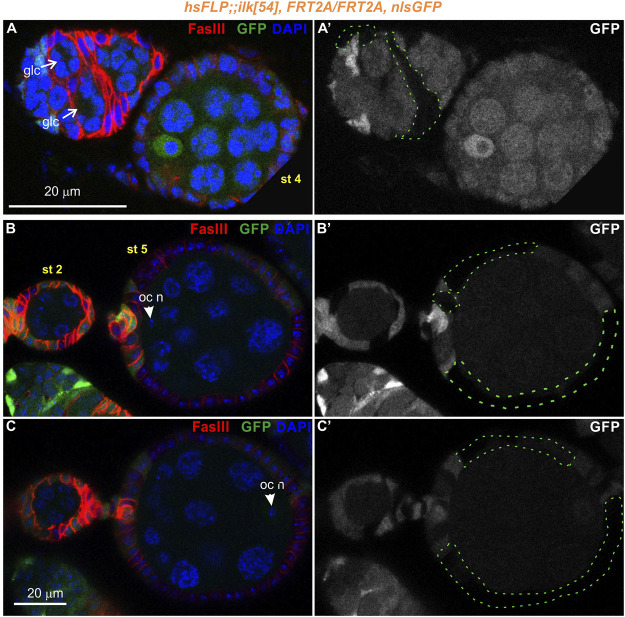
Genetic mosaics for *Ilk* display germline cyst encapsulation defects resulting in compound egg chambers. **(A–C)** Confocal micrographs of egg chambers carrying *Ilk* mutant clones were generated by *hsFLP* and marked by the absence of the nlsGFP. The presence of the mutant clones are highlighted with a green dashed line. Co-staining with anti-FasIII labels the periphery of follicle epithelial cells. **(A-A′)** A pair of germline cysts in region 2b of the germarium surrounded by *Ilk* mutant cells. **(B,C)**. Different optical sections of the same egg chambers reveal the compound egg chamber at stage 5, which contains two individual oocyte nuclei. **(B-B′)** An oocyte nucleus is located in the anterior end of the compound egg chamber. **(C-C′)** The second oocyte nucleus is located in the opposing posterior end of the same compound egg chamber. glc, germline cyst; oc n, oocyte nucleus. Scale bars: 20 μm in all panels.

To analyze further the functional requirement of Parvin in egg chamber morphogenesis we initially generated genetic mosaics for *Parvin* using two Gal4 drivers: a) *e22cGal4* driver, which is expressed in the escort cells in the anterior part of the germarium, in the follicle stem cells at the interface between regions 2a and 2b and in almost all follicle epithelial cells after stage 2 (Figure 7A, Figure 7C-C′) and (Duffy et al., 1998); b) *bab1Gal4* driver, which is expressed in the terminal filament cells in the anterior part of the germarium, in the posterior pre-follicle cells of the germarium, in polar cells and a large fraction of follicle epithelial cells after stage 2 (Figure 7B, Figure 7D-D′) (Cabrera et al., 2002). We previously confirmed that *Parvin* ^*A*^, *FRT19A* allele is a null allele, like *Parvin* ^*694*^ (Vakaloglou et al., 2012; Yamamoto et al., 2014) and therefore we used it in our clonal analysis. We confirmed that loss of Parvin from the cells expressing *e22cGal4* and *bab1Gal4* leads to defects in germline cyst encapsulation in the developing germaria (Figures 7E–G). Double-containing cysts in the germarium were observed when *Parvin* mutant cells encircled each germline cyst (n = 12/61 mosaic germaria containing a very large Parvin mutant clone) (Figures 7E–G). Similarly, we found that heat shock-induced *Ilk* mutant clones lead similarly to double germline cysts in region three of the germarium (Figure 8A). However, the recovery of mosaic germaria containing heat shock-induced large *Ilk* clones in pre-follicle cells was relatively low (*n* = 2/19 mosaic germaria). Collectively, our data suggest that both Parvin and ILK are required in the migrating pre-follicle cells to encapsulate the developing cysts in the germarium.

**FIGURE 9:**
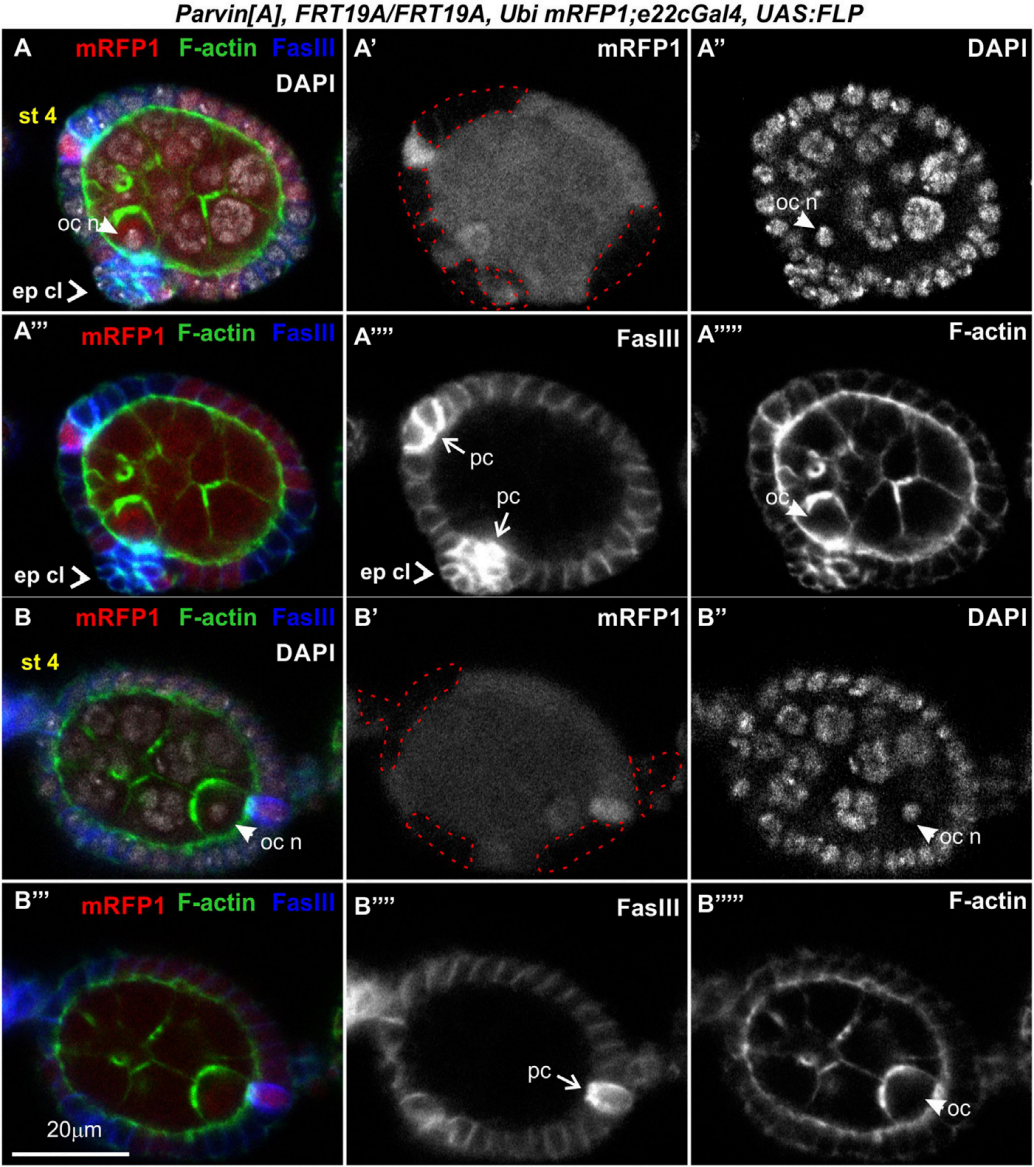
Genetic mosaics for *Parvin* generated with *e22cGal4* lead to epithelial clusters and fused egg chambers. **(A-B‴'')** Confocal micrographs of a stage 4 egg chamber derived from *Parvin* ^*A*^ mosaic flies generated by *e22cGal4*. Co-staining with FasIII and F-actin labels the periphery of follicle epithelial cells. **(A,B)** A compound egg chamber at different optical cross sections encircled by mutant cells for *Parvin*. **(A′,B′)** *Parvin* ^*A*^ mutant cells were marked by the absence of *mRFP1* and are highlighted with a red dashed line. **(A-A‴'')** Protruding epithelial clusters in the middle region of the compound egg chamber characterized by elevated FasIII protein levels. **(A′,A‴')** The anterior polar cells are located in the same focal plane with the protruding epithelial cluster. The oocyte nucleus of the younger cyst is localized laterally of the epithelial protruding cells. **(A")** The oocyte nucleus of the younger cyst is located laterally of the epithelial protruding cell cluster. **(B-B‴'')** The oocyte nucleus of the older cyst is located in **(B′)** the posterior end and in close contact with **(B‴')** the posteriorly located polar cells. ep cl, epithelial cluster; oc, oocyte; oc n, oocyte nucleus; pc, polar cells. Scale bars: 20 μm in all panels.

**FIGURE 10:**
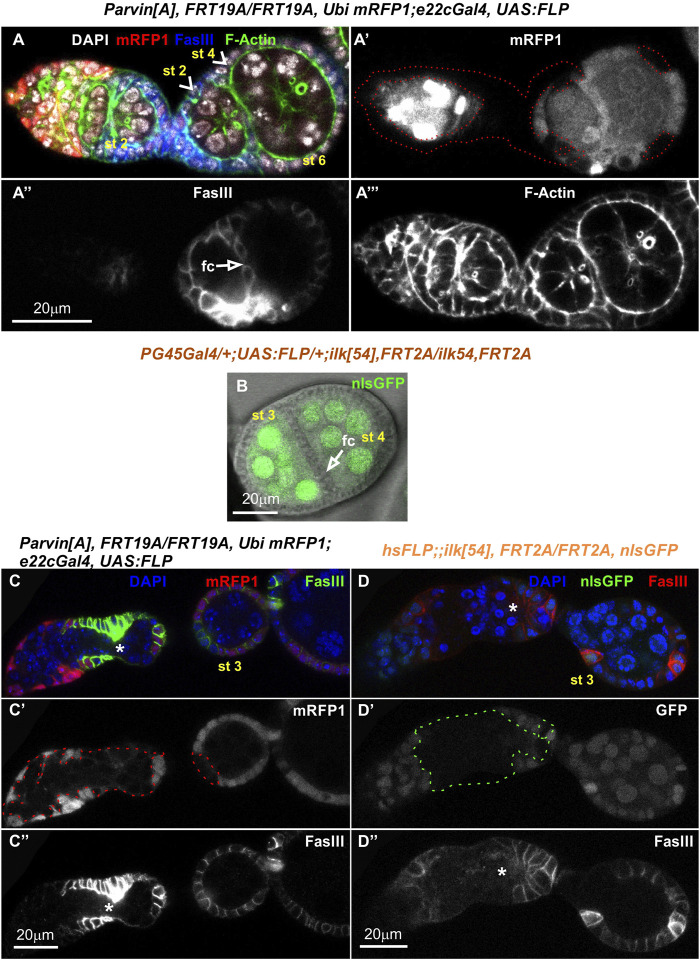
Genetic mosaics for *Parvin* and *Ilk* lead to fused or malformed egg chambers. Confocal micrographs of egg chambers derived from **(A-A‴,C-C")** *Parvin* ^*A*^ mosaic adult fly generated by *e22cGal4* and **(B,D-D")** *Ilk* ^*54*^ mosaic adult fly generated by **(B)** *PG45Gal4*, which starts to express very early in oogenesis in almost the entire somatic epithelium resulting in very large mutant clones or by **(D-D")** *hsFLP*, which is expressed in a stochastic manner and less frequently produces large clones. Fused *Parvin* mosaic egg chambers (st2-st4) separated by a monolayer of epithelial cells. Co-staining with F-actin and FasIII labels the periphery of follicle epithelial cells. **(A′,C′)** *Parvin* ^*A*^ mutant cells were marked by the absence of *mRFP1* and highlighted with a red dashed line. **(B)** Merged confocal fluorescence and phase contrast microscopy image depicts fused *Ilk* egg chambers (st3-st4) separated by a monolayer of epithelial cells. *Ilk* ^*54*^ mutant cells were marked by the absence of *nlsGFP*. **(C,D)** No obvious separation between the distinct stages of the germarium in **(C-C")** *Parvin* ^*A*^ mutant clone and **(D-D")** *Ilk* ^*54*^ mutant clone. Asterisks indicate the lack of separation between R2 and R3 in the germarium. fc, follicle cells. Scale bars: 20 μm in all panels.

**FIGURE 11:**
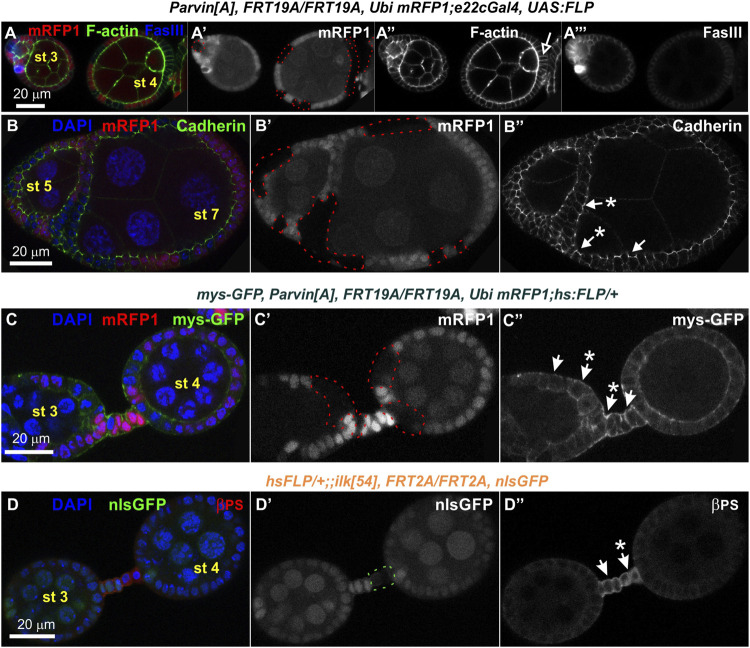
Loss of Parvin or ILK does not cause multilayering of the terminal follicle epithelium and does not affect the localization of cadherins and integrins. Confocal micrographs of egg chambers derived from **(A-B")** *Parvin* ^*A*^ adult mosaic flies generated by *e22cGal4* **(C-C")** *Parvin* ^*A*^ adult mosaic flies generated by *hsFLP* with co-expression of mys-GFP to monitor βps integrin endurance and **(D-D")** *ilk* ^*54*^ adult mosaic flies generated by *hsFLP.* **(A-A")** Follicle cells mutant for *Parvin* positioned at the edges of the egg chamber do not create layering defects. Co-staining with F-actin and FasIII labels the periphery of follicle epithelial cells. **(B-B")** Cadherin is properly expressed and localized at the apical adhesion sites in *Parvin* ^*A*^ mutant cells. **(C-C′)** The endogenous expression and localization of *mys-GFP* remains unaffected in *Parvin* ^*A*^ mutant cells **(A′–C′)** *Parvin* ^*A*^ mutant cells were marked by the absence of the mRFP1 and are highlighted with a red dashed line. **(D-D")** Co-staining against β_PS_ integrin reveals that integrin expression and localization in pre-follicle and stalk cells does not require ILK function. **(D′)** *Ilk* ^*54*^ mutant cells were marked by the absence of the *nlsGFP* and are highlighted with a green dashed line. Asterisks indicate mutant cells. Scale bars: 20 μm in all panels.

Interestingly, in subsequent developmental stages egg chambers that contained either *Parvin* (*n* = 26/193 mosaic egg chambers) or *Ilk* mutant clones (*n* = 2/12 mosaic egg chambers) were compound. A compound egg chamber refers to a phenotype that arises when two neighbouring germline cysts are enveloped together in one egg chamber (Hawkins et al., 1996; Jackson and Blochlinger, 1997). The compound egg chambers were formed even when a fraction of follicle cells were mutant for *Parvin* (Figures 7F,G) or *Ilk* (Figures 8B,C). Two oocyte nuclei were positioned opposite at the two ends of the compound egg chamber, together with larger size nuclei of 25–30 nurse cells (Figure 7G; Figure 8B,C. Based on elevated FasIII levels these compound egg chambers contain invariable number of polar cells in four distinct locations within the follicular epithelium (Figure 9, Supplementary Figures S3, 4). In a large fraction of the analyzed compound egg chambers containing *Parvin* mutant clones (*n* = 21/26), we identified epithelial cells arranged as clusters and ectopically protruding in the middle of the egg chamber (*n* = 21/21) (Figures 9A′′′′, 9B′′′′). These clustered cells, likely constitute a mixture of polar cells-because they expressed high levels of FasIII-and stalk cells that have failed to form an interfollicular stalk. We also found compound egg chambers, where the polar cells were ectopically located in the basal side of the egg chamber (Supplementary Figures S4A′′–D′′). The anteriorly located oocyte nucleus was mispositioned laterally along the anterior-posterior axis and in contact with the cluster of epithelial cells (Figures 9A,A′′′,A′′′′). We also identified egg chambers containing either *Parvin* (*n* = 45/193) or *Ilk* (*n* = 3/12) mutant clones and the two successive egg chambers were attached without an interfollicular stalk, leading to end-to-end fusions (Figures 10A,B). Finally, in mosaic egg chambers containing either *Parvin* (*n* = 56/137) or *Ilk* (*n* = 9/16) mutant clones in the germarium, we identified a lack of separation between R2 and R3 regions (Figures 10C,D). We also examined whether the loss of either Parvin or ILK cause other integrin related phenotypes in the developing egg chambers. Terminal *mys* integrin clones cause epithelial multilayering (Fernández-Miñán et al., 2007). However, removal of Parvin (*n* = 0/33 egg chambers containing terminal mutant clone) or ILK (*n* = 0/6 egg chambers containing terminal mutant clone) from the posterior terminal follicle epithelial cells did not cause abnormal epithelial multilayering (Figure 11A). Finally, we examined whether the localization of apical or basal adhesion components is affected in *Parvin* or *Ilk* mutant clones. The cadherin apical-lateral localization was not affected, suggesting that apical cell-cell junctions remained intact in *Parvin* mutant cells (Figure 11B). Similarly, there was no disturbance in the expression and localization of β_PS_ integrin in *Parvin* (Figure 11C) and *Ilk* (Figure 11D) mutant clones in pre-follicle and stalk cells. Thus, the defects observed in the loss of Parvin and ILK are not related to the abnormal expression or distribution of the two main cell adhesion components.

### Genetic mosaics for parvin confirm its essential role in interfollicular stalk formation

**FIGURE 12:**
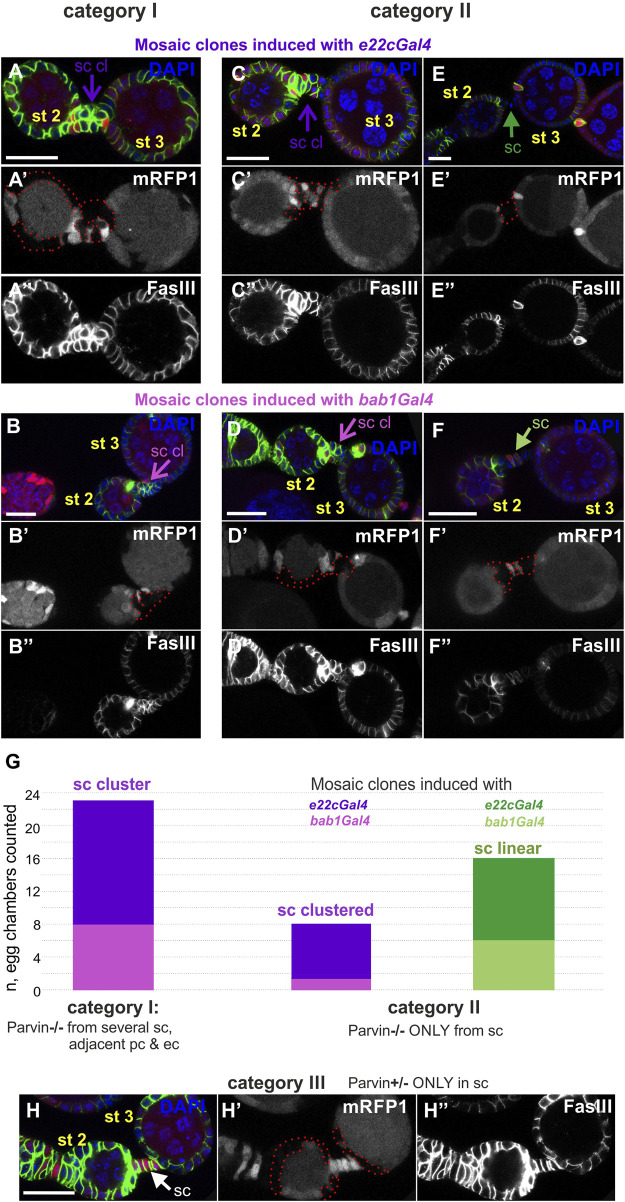
Cell-autonomous and non-cell-autonomous effect of Parvin genetic mosaics in the interfollicular stalk formation. Confocal micrographs of early stage (st2 to st3) egg chambers derived from **(A,E")** *Parvin* ^*A*^ adult mosaic flies generated by *e22cGal4* **(B,F")** *Parvin* ^*A*^ adult mosaic flies generated by *bab1Gal4*. **(A′–F′)** *Parvin* ^*A*^ mutant cells were marked by the absence of the *mRFP1* and are highlighted with a red dashed line. Co-staining with FasIII labels follicle epithelial cells. **(A,B")** Parvin mutant clones generated with **(A-A")** *e22cGal4* or **(B,B")** *bab1Gal4* in stalk cells and in adjacent polar and follicle epithelial cells **(G)**, category I, *n* = 23/23) lead to stalk cell clusters. **(C,F")** Stalk cells form clusters in between egg chambers less often (G, category II, *n* = 8/23), although several of them contain *Parvin* mutant clones but the adjacent polar and follicle epithelial cells express endogenous Parvin. Mosaics are again generated with either **(C-C")** *e22cGal4* or **(D-D")** *bab1Gal4* but result in clone formation exclusively in stalk cells and not in the adjacent polar and follicle epithelial cells. **(H-H")** *Parvin* mutant clones generated with *e22cGal4*, exclusively in the adjacent polar and follicle epithelial cells, while stalk cells contain Parvin, lead to proper stalk cell arrangement in a linear order (category III, *n* = 0/4). sc: stalk cell; sc cl, stalk cell cluster Scale bars: 20 μm in all panels.

We additionally analyzed the stalk cell organization by examining a variety of mosaic combinations within the developing egg chambers, using either *e22cGal4 or bab1Gal4*. When Parvin was missing both from several stalk cells and the adjacent polar and follicle epithelial cells, the stalk cells invariably formed clusters (category I, n = 23/23, Figures 12A,B,G). When Parvin was missing only from several stalk cells, while the adjacent cells contained Parvin, clusters were less often formed (category II, *n* = 8/23, Figures 12C–G). Surprisingly though, when Parvin was present only in stalk cells but it was missing from both the adjacent polar and follicle epithelial cells, the stalk cells were properly arranged in a linear order (category III, *n* = 0/4, Figure 12H). In contrast, in the data obtained with the *24BGal4* rescued *Parvin* ^*694*^ stalk cells formed clusters although Parvin-GFP was expressed in fully differentiated stalk cells from stages 2–3, but it was not expressed in the adjacent polar and follicle cells (Figures 6D,E). From these data, we concluded that Parvin is required for stalk cell morphogenesis in an autonomous and non-autonomous manner, while the timing of clone formation is critical.

## Discussion

The developing *Drosophila* egg chamber provides a very attractive model system to study epithelial patterning and morphogenesis (Horne-Badovinac and Bilder, 2005; Osterfield et al., 2017). The follicular epithelium that encircles the germ cells undergoes a series of morphogenetic events including cell proliferation, collective cell movement and cell intercalation. These particular steps in epithelial morphogenesis are tightly coordinated with the growth of the encompassed oocyte and require modulation of cell-cell and cell-matrix adhesion (McCaffrey et al., 2006; Bergstralh et al., 2015; Reilly et al., 2015; Chanet and Huynh, 2020). The role of integrin-mediated cell-matrix adhesions was recently elucidated in the pre-follicle cells (Lovegrove et al., 2019). In the present study, we show that elimination of Parvin and ILK from pre-follicle cells phenocopy the removal of integrins, although there are penetrance and expressivity differences. We found that germline cyst encapsulation and interfollicular stalk formation require Parvin and ILK in the pre-follicle cells. In contrast, the preservation of the monolayer organization in the middle stage egg chambers termini although it requires integrins (Fernández-Miñán et al., 2007; Lovegrove et al., 2019), it does not require Parvin or ILK. Collectively, our data provide novel evidence for the molecular machinery that is required downstream of integrins to couple follicle cells’ intrinsic signals with the spatial cues of the extracellular microenvironment and thus drive epithelial morphogenesis.

### Parvin and ILK functional requirements in the pre-follicle cells

We initially determined the spatiotemporal protein expression of Parvin and ILK, using the genomic translational fusion rescue constructs tagged with GFP for each gene. Both transgenes were expressed under their endogenous regulatory elements (Zervas et al., 2001; Vakaloglou et al., 2012). Parvin and ILK displayed an identical expression pattern, low levels in the follicle epithelial cells, but accumulate at significantly higher levels in the terminal filaments, cap cells and interfollicular stalks. Because both *Parvin* and *Ilk* null mutant alleles are embryonic lethal, we circumvent the earlier developmental requirement for both genes by utilizing a dual genetic strategy. First, we took advantage of the ability to fully rescue the embryonic lethality to adult viability for each mutant allele by just expressing the relevant wild-type UAS transgene with the *24BGal4* driver (Zervas et al., 2001; Vakaloglou et al., 2012). With this approach, we were able to remove throughout oogenesis either Parvin or ILK from the follicle epithelial cells, besides the terminal filament and the stalk cells. Second, we generated marked mitotic clones utilizing the FLP/FRT system (Xu and Rubin, 2012). Both approaches allowed us to identify the essential requirement for Parvin and ILK in the early stages of germaria development, and further identify that the time-dependent removal of Parvin and ILK is critical for the observed defects. Initially, we identified that loss of Parvin or ILK resulted in defective germline cyst encapsulation. Similarly, knockdown of *mys* with the *TJGal4* resulted in incomplete encapsulation and fusion of the germline cysts (Lovegrove et al., 2019). Proper germline cyst encapsulation is a prerequisite for the subsequent egg chamber development and previous genetic studies have illustrated that abnormalities in cyst encapsulation can arise as an outcome of several defective processes. For example in DLar mutants, the two cysts in region 2b fail to modify their shape (Frydman and Spradling, 2001). In absence of *brainiac* (*brn*) or *egghead* (*egh*), which both encode glycosyltransferases and are required in the germline, pre-follicle cells fail to recognize the boundary of the individual cysts and migrate between them (Goode et al., 1996; Horne-Badovinac and Bilder, 2005). Furthermore, in *Stat92E* mutants germline cysts fail to properly encapsulate and are frequently accompanied with lack of separation between regions R2-3 in the germarium (Baksa et al., 2002). Recently it was shown that integrins (*mys*) are required in the germarium to maintain the attachment of the pre-follicle cells to the ECM during the germline cyst encapsulation (Lovegrove et al., 2019). Thus, one explanation could be that Parvin and ILK loss-of-function destabilize the integrin-ECM adhesion. Consequently, the pre-follicle cells fail to extend and occupy the entire area covering the newly formed germline cysts, leaving available space for the next cyst to enter region 2b. Furthermore, mutants in the gene *cheerio*, which encodes the actin-binding protein filamin disrupt the formation of cellular extensions in the pre-follicle cells, leading to migration defects (Sokol and Cooley, 2003). Interestingly, it has been shown that Parvin and ILK control the formation of filopodia by blocking the cofilin-mediated F-actin severing in metastatic cancer cells (Shibue et al., 2013). This fits with the requirement of cofilin in cell motility during ovary development (Chen et al., 2001). The recent identification of the actin-binding WH2 motif in Parvin further suggests that Parvin and ILK may be implicated in the fine-tuning of the F-actin structures (Vaynberg et al., 2018). Thus, we envisage that Parvin and ILK could disrupt germline encapsulation either due to the inability of pre-follicle cells to remain strongly attached to the basement membrane surrounding the germline, or/and due to disturbances in actin cytoskeleton reorganization, which decrease the motility of the pre-follicle cells during encapsulation.

Loss of β_PS_ from stalk cells and neighbouring follicle cells affects interfollicular morphogenesis without affecting the differentiation of pre-follicle cells to stalk cells, as Lamin C and *24BGal4* are still expressed in mutant cells of the disorganised stalks. Instead, the loss of integrins, ECM components or Tensin affect the process of stalk cell intercalation leading to morphological abnormalities (Cha et al., 2017; Lovegrove et al., 2019; Van De Bor et al., 2021). Similarly, we found that Parvin and ILK are required in the formation of the interfollicular stalks, without affecting stalk cell differentiation. However, we obtained some contradictory results between our clonal analysis and *24BGal4*-mediated rescue of the *Parvin* and *Ilk* mutants. In *24BGal4* rescued egg chambers there was a fully penetrant interfollicular stalk defective phenotype, despite the expression of the UAS:Parvin-GFP or UAS:ILK in the fully differentiated stalk cells (Figures 6D–F). Instead of four to eight cells that typically form the interfollicular stalk, there were 12–16 cells that formed a cluster, suggesting that these cells have lost their capacity to converge and form a one-layer stalk. Similarly, we always recovered defective interfollicular stalks when several stalk cells and the adjacent polar and epithelial cells were mutants (Figures 12A,B, category I). One possibility is that the surrounding mutant epithelial follicle cells non-autonomously contribute to interfollicular stalk formation. However, when *Parvin* mutant clones were recovered only in the follicle epithelial cells, while the stalk cells were wild-type, the interfollicular stalks were properly organized (Figure 12H, category III). The discrepancy in our obtained results perhaps is related to the temporal differences in the generation of the abnormally organized follicle tissue, which contains a mixture of mutant and non-mutant cells. *24BGal4* starts to express in the pre-stalk cells right after stage 2 of oogenesis (Figure 5C), while the critical time of integrin requirement was shown to be before stage 2/3 (Lovegrove et al., 2019). Thus, similar to integrins the functional requirement of Parvin and ILK is correlated with the absence of *24BGal4* expression from the pre-stalk cells. However, it is not clear whether the pre-stalk cells had efficiently depleted *Parvin* during intercalation when the mosaic clones were generated with either *e22cGal4* or *bab1Gal4*. Collectively, we concluded that interfollicular stalks depend on Parvin and ILK in a cell-autonomous and non-autonomous manner and there is a critical timing of their requirement, presumably when pre-follicle cells encircle the germ cells in the germarium.

In the genetic mosaics, we frequently found compound egg chambers containing two germline cysts encircled by a single epithelial layer or fused egg chambers without an interfollicular stalk. We found two features of defective cell intercalation. First, we found protruding cell clusters in the *24BGal4* rescued *Parvin* and *Ilk* mutants. These clusters were frequently ectopically positioned laterally, maintaining though contact with the two consecutive egg chambers, while *24BGal4* expression identifies them as stalk cells. Second, we also found ectopically positioned clusters of epithelial cells in the proximity of the anterior oocyte. These clusters were constituted from polar cells and likely stalk cells, and formed a bulge protruding out of the egg chamber (Torres et al., 2003). We concluded that the observed clustered cells represent incomplete intercalation within follicular epithelium. In contrast to the talin-loss-of function mutants where cadherin expression is decreased in an integrin-independent manner, (Bécam et al., 2005), loss of Parvin and ILK do not affect cadherin or integrin expression.

In summary, our study uncovers the essential functional requirement of ILK and Parvin-two core components of the integrin adhesome-in epithelial morphogenesis and tissue architecture preservation, presumably by integrating extracellular cues to integrins and actin cytoskeleton.

## Materials and methods

### *Drosophila* genetics

The following *Drosophila* mutant alleles were used: *Parvin* ^*A*^ (Yamamoto et al., 2014), *Parvin* ^*694*^ (Vakaloglou et al., 2012), *ilk* ^*54*^ (Zervas et al., 2011), *ilk* ^*1*^ (Zervas et al., 2001), *Parvin-GFP* (Vakaloglou et al., 2012), *ILK-GFP* (Zervas et al., 2001) and *mys-GFP* (Klapholz et al., 2015). *UAS:Parvin-GFP* (Vakaloglou et al., 2012) and *UAS:ILK* (Zervas et al., 2001) were expressed under the control of *24B-Gal4* driver (Brand and Perrimon, 1993). To generate somatic mutant clones we used the FRT/FLP technique (Chou and Perrimon, 1992). The *Parvin* ^*A*^ *, FRT19A* clones were generated with the use of *bab1-Gal4* driver (Cabrera et al., 2002; Bolivar et al., 2006) and *e22c-Gal4* driver (Duffy et al., 1998). The *Ilk* ^*54*^ *, FRT2A* clones were generated either by *hsFLP* (BL-7) or *UASFLP* driven by *PG45Gal4* and were marked by the absence of *nlsGFP*. The *PG45Gal4* driver, kindly provided by Dr Ellen LeMosy starts to express very early in oogenesis in almost the entire somatic epithelium (Zappia et al., 2011). The *Parvin* ^*A*^ *, FRT19A/FM7-eGFP* females were crossed with *w, Ubi mRFP1 nls,FRT19A; e22c-Gal4,UAS-FLP* males in order to produce mosaic egg chambers for *Parvin* mutant cells,. *Parvin* mutant clones were marked by the absence of *mRFP1*.

### Immunohistochemistry, microscopy and image analysis

*Drosophila* larval ovaries were dissected according to the protocol (Maimon and Gilboa, 2011). Adult ovaries dissection was done according to the protocol (Wong and Shedl, 2011). Primary antibodies used in this study were: mouse monoclonal anti-βPS (Developmental Studies Hybridoma Bank *CF.*6G11; 1:10), mouse monoclonl anti-FasIII (Developmental Studies Hybridoma Bank 7G10; 1:20), rat monoclonal anti-DE- Cadherin (Developmental Studies Hybridoma Bank DCAD2; 1:20), 1:500, rabbit polyclonal anti-PAK (kind gift from Dr Harden) (Vlachos and Harden, 2011) and rabbit polyclonal anti-VASA at 1/5,000. Species-specific secondary antibodies used were conjugated with AlexaFluor 488, 568 or 633 (Molecular Probes by Life Technologies) and Cy3 or Cy5 (Jackson ImmunoResearch Laboratories) diluted at 1:1,000. Nuclei were labelled with DAPI. F-actin was visualized using either Rhodamine-Phalloidin or AlexaFluor 647-Phalloidin at 1:500 dilution (Molecular Probes, LifeTechnologies). All samples were mounted in Vectashield medium (Vector Laboratories). The fluorescent intensity of *Parvin-GFP* and *Ilk-GFP* in egg chambers was measured in several single confocal sections. Mean value of fluorescence intensity of manually selected areas of the same size was quantified using ImageJ software.

Single confocal sections and z stacks were acquired on a Leica TCS SP5 laser scanning inverted confocal microscope with an HC Plan Apochromat 20x/0.7 or HC Plan-Apochromat 63x/1.4 oil objective. Confocal settings were adjusted to avoid pixel intensity saturation of 1,024 × 1,024 pixel images captured at 400 Hz. Post-acquisition assembly was performed with LAS AF software (v.2.3.6). 3D rendering of confocal images was generated using the Volocity software. Images were assembled in Photoshop seven and labeled in Corel Draw 12.

### Fecundity assay

Fecundity assay was performed in order to evaluate the egg-laying capacity per day of tissue-specific rescued females for either Parvin or ILK (Hanson and Ferris, 1929; Chapman and Partidge, 1996). 25 newly hatched females *Parvin* ^*694*^/*Parvin* ^*694*^; *UAS:Parvin-GFP*/*24BGal4* and *UAS:ILK/+;ilk* ^*54*^ *,24BGal4/ilk* ^*1*^ were crossed for 2 days with wild type males. On the third day, flies were transferred in a custom made cage with an agar juice plate at the bottom supplied with fresh yeast paste where females deposit their eggs. During the day the agar juice plate with the yeast was replaced every 3 h and the embryos in the old agar juice plate were counted. The duration of the fecundity assay was 4 days. At the same time, a fecundity assay was performed in *UAS:Parvin-GFP/24BGal4, UAS:ILK/+;24BGal4* and *24BGal4/24BGal4* females as control.

## Data Availability

The original contributions presented in the study are included in the article/Supplementary Material, further inquiries can be directed to the corresponding author.
